# Investigating Oral Health Information Avoidance Among Chinese Young Adults: The Roles of Subjective Norms, Message Fatigue, and Loss Aversion—A Cross-Sectional Study

**DOI:** 10.3390/bs16050704

**Published:** 2026-05-04

**Authors:** Dehuan Liu, Donghwa Chung, Yanfang Meng, Jiaqi Wang

**Affiliations:** 1 College of Media and Communication, Peking University, Beijing 100871, China; dehuanliupku@outlook.com; 2 School of Journalism and Communication, Central China Normal University, Wuhan 430079, China; 3 School of Journalism and Communication, Beijing Institute of Graphic Communication, Beijing 102699, China; mengyf@bigc.edu.cn; 4 School of Journalism and Communication, Xiamen University, Xiamen 361005, China; jiaqiwang@stu.xmu.edu.cn

**Keywords:** oral health, health information avoidance, prospect theory, short-form video, message fatigue, survey

## Abstract

As media users increasingly engage in defensive avoidance or disengagement from health-related content, oral health information has attracted growing attention, particularly given the tendency of Chinese young adults to avoid such content. This avoidance may constrain oral health awareness and related practices. Guided by Prospect Theory, the current study examines the association between engagement with oral health short videos and oral health information avoidance among Chinese young adults, and further investigates the mediating roles of subjective norms and message fatigue, as well as the moderating role of loss aversion. Data were collected from 10 December 2025, to 10 February 2026, via an online questionnaire, yielding 306 valid responses from Chinese young adults aged 18–35. All variables were measured using five-point Likert scales. Analyses were conducted using Jamovi 2.6.44 and SPSS 29.0. Reliability and validity were assessed, and direct and indirect associations, as well as moderation effects, were examined using bootstrapping with 2000 resamples. Engagement with oral health short videos was positively associated with message fatigue. Message fatigue mediated the association between engagement and oral health information avoidance. In addition, loss aversion moderated the relationship between message fatigue and oral health information avoidance. Health promoters (i.e., content creators) and oral health professionals should adopt targeted strategies to enhance communication effectiveness. Excessive algorithm-driven exposure that is misaligned with users’ needs should be minimized. Incorporating gain-framed and supportive messaging may reduce perceived pressure and improve receptivity to oral health content.

## 1. Introduction

Oral health has emerged as a major global public health concern, with oral diseases affecting approximately 3.7 billion people worldwide and nearly half of the population experiencing at least one condition each year (Franklin, 2024; WHO, 2025). Many oral diseases, including dental caries, periodontal disease, tooth erosion, and oral cancer, are chronic and require complex, long-term management, contributing to a substantial and persistent burden (W. S. H. Chan, 2024; Nuriddinovna, 2025; Patterson-Norrie et al., 2023). Despite increasing attention, multiple barriers continue to hinder effective oral healthcare utilization, including clinical complexity, limited access to timely services, dental anxiety, high treatment costs, and inadequate oral health literacy (Felgner et al., 2022; S. Yu et al., 2024; Zammit, 2024). These factors delay appropriate care-seeking, exacerbate disease progression, and underscore the need for more effective interventions.

Given the substantial global burden and wide-ranging health consequences of oral diseases, coordinated policy and communication initiatives have been widely implemented worldwide to advance oral health (Anyikwa & Ogwo, 2025). China has notably elevated oral health promotion as a public health priority, emphasizing improvements in oral health literacy and the adoption of preventive practices (TX, 2025). With the implementation of national digital health initiatives, online communication has demonstrated substantial potential for advancing oral health promotion. Recent evidence suggests that mass media serve as a primary channel for oral health campaigns in China (Goldberg et al., 2022). As social media have become increasingly influential, short-form video platforms have emerged as effective channels for delivering oral health information to the public (Y. Sun, 2025) and facilitating user engagement with health-related content (ZGQN, 2024). Chinese young adults actively like, comment on, and share oral health short videos, reflecting growing engagement with oral health content.

Although social media-based oral health promotion, particularly via short-form video platforms, has shown promise in improving public awareness and engagement in China (Huang et al., 2025; S.-Y. Zhang et al., 2024), such initiatives may also trigger health information avoidance across promotion contexts. For instance, some Chinese young adults share on social media that they intentionally ignore dental discomfort and avoid others’ suggestions for treatment (ZT, 2023). Health information avoidance is conceptualized as a maladaptive coping behavior associated with delayed clinical intervention and adverse health outcomes (Howell et al., 2020; Sweeny et al., 2010). Conceptually, information avoidance functions as a psychological mechanism through which individuals intentionally disengage from threatening or overwhelming health information, thereby potentially undermining their capacity for effective health self-management (Howell & Shepperd, 2017; Ranjbar et al., 2023). Empirical evidence of health information avoidance has been identified across multiple health contexts, including COVID-19-related information (Mao et al., 2024; Soroya et al., 2021), cancer-related information (Zhu et al., 2024), and dementia-related information (Orom & Allard, 2025).

Building on this line of research, scholars have examined factors that drive media users to disengage from health information, including demographic characteristics (Howell & Shepperd, 2017) and affective concerns (Persoskie et al., 2014). More recent studies indicate that social media use, together with cognitive, emotional, and normative factors, is associated with health information avoidance on digital platforms (Ahn & Kahlor, 2023; So et al., 2017). For instance, Schumann (2022) found that extensive media exposure to COVID-19-related content can induce issue fatigue, which may subsequently lead to information avoidance (Schumann, 2022). In this context, engagement with short-form video platforms in China may similarly contribute to the avoidance of oral health information.

This study draws on Prospect Theory and integrates key psychological and normative antecedents to examine the relationship between engagement with oral health short videos (EOHSV) and oral health information avoidance (OHIA) among Chinese young adults aged 18–35 years. It further examines the underlying mechanisms of this association to address gaps in the existing literature. This study provides both theoretical contributions and practical implications for health communication research and intervention design.

## 2. Literature Review

### 2.1. Prospect Theory

Prospect Theory, originally proposed by psychologists Kahneman and Tversky, explains how individuals make decisions under conditions of risk and uncertainty (Kahneman & Tversky, 2013). The theory describes the cognitive process through which individuals evaluate potential outcomes as gains or losses relative to a reference point (e.g., loss aversion, status quo), rather than based on their absolute final outcomes (Kahneman & Tversky, 2013). Furthermore, it has been proposed that when individuals are presented with equivalent options, they tend to assign greater psychological weight to losses than to gains and prioritize avoiding losses over achieving comparable gains, a phenomenon known as loss aversion (Gal & Rucker, 2018). Building on this concept, prior research has explored individuals’ risk-taking behaviors from a loss aversion perspective, demonstrating that those with stronger loss aversion tend to adopt more conservative decision-making strategies under conditions of uncertainty (Arora & Kumari, 2015; Gupta & Shrivastava, 2022).

A growing body of research in the field of health communication has examined loss aversion in health decision-making, showing that individuals often perceive changes to their current situation as potential losses (Lam & Lin, 2025). For example, individuals may avoid health screening to prevent anticipated psychological or practical losses when facing the risk of chronic disease (Adonis, 2015). Cancer-related fear has similarly been linked to heightened loss aversion and increased information avoidance (Lam & Lin, 2025). Given that loss aversion leads individuals to overweight the perceived costs of recommended health practices, this study examines its role in shaping oral health information avoidance among Chinese young adults. Although guided by Prospect Theory, the present study focuses on loss aversion as an individual difference factor, reflecting the tendency to weigh potential losses more heavily than equivalent gains, which may influence information avoidance.

### 2.2. Mediation Role of Subjective Norms

The Theory of Planned Behavior (TPB) posits that attitudes, subjective norms, and perceived behavioral control jointly shape individuals’ behavioral intentions and subsequent actions (Ajzen, 1991). To better understand mechanisms underlying conscious decision-making, numerous researchers have applied TPB across diverse contexts, including preparedness for future earthquake disasters (Zaremohzzabieh et al., 2021), impulsive buying behaviors (Y. Liu et al., 2023), and health information sharing (Wu & Kuang, 2021). Among the core constructs of TPB, subjective norms have emerged as a particularly influential predictor of health-related behaviors (Yarmohammadi et al., 2023). Subjective norms refer to individuals’ perceptions that important others expect them to perform a particular behavior and believe they should engage in it (Li et al., 2023). In this study, subjective norms are conceptualized as perceived social expectations to disengage from oral health-related content, reflecting a context-specific adaptation of TPB that extends the construct from behavioral engagement to information avoidance.

Social norms have been shown to exert a stronger influence on the adoption of recommended behaviors (e.g., pro-environmental actions) than individually obtained information, reflecting the prioritization of group harmony over personal preferences in collectivist societies (Qiu, 2025). Consistent with this perspective, subjective norms have been widely examined in China, with prior studies demonstrating their significant influence across various health-related behaviors, including HPV vaccination (Shi et al., 2025), COVID-19 prevention (Duan et al., 2022), healthy eating (Xu & Hwang, 2025), and the use of traditional Chinese medicine (Ng et al., 2022).

A growing body of media effects research indicates that individuals’ engagement with media can induce subjective norms. For example, individuals frequently exposed to anti-alcohol and anti-marijuana content are more likely to report stronger subjective norms (Cristello et al., 2024). In the context of health misinformation, engagement with misleading content has similarly been found to be strongly associated with subjective norms (Oktavianus & Bautista, 2023). In addition, subjective norms regarding green product purchases have been identified as a key outcome influenced by individuals’ social media use (Kumar & Pandey, 2023). Therefore, the following hypothesis is proposed.

**Hypothesis** **1.** *EOHSV is positively associated with subjective norms among Chinese young adults.*

Growing evidence indicates that media engagement is indirectly associated with behavioral intentions. For instance, individuals’ perceptions of social network support for COVID-19 vaccination (i.e., subjective norms) have been shown to increase the likelihood of adopting preventive behaviors (Strathdee et al., 2023). A systematic review likewise identified subjective norms as a key determinant of exercise intentions (Wang & Ali, 2025). However, subjective norms do not consistently facilitate behavior adoption across contexts. Empirical evidence further suggests that, in certain situations, subjective norms may evoke defensive psychological responses. For example, subjective norms were found to be negatively associated with individuals’ intention to adopt a digital platform (Shittu & Salisu, 2023). Prior research similarly found no significant association between subjective norms and rural Iranian women’s breast cancer screening intentions (Keshavarzi et al., 2022). Given these mixed findings, it is plausible that frequent EOHSV may shape Chinese young adults’ normative beliefs and perceptions that avoidance of oral health information is socially acceptable, thereby reinforcing avoidance-oriented attitudes and ultimately increasing OHIA. Accordingly, the present study proposes the following mediation hypothesis.

**Hypothesis** **2.** *Subjective norms mediate the association between EOHSV and OHIA among Chinese young adults.*

### 2.3. Mediation Role of Message Fatigue

A growing body of research has examined the adverse psychological consequences of social media engagement. Among these, message fatigue has emerged as a common experience among media users (Ou et al., 2023). Message fatigue refers to a state of exhaustion and boredom resulting from repeated exposure to similar messages over time (So et al., 2017). Previous studies have examined the direct relationship between social media use and message fatigue. For example, Hickerson and Stamps found that greater social media consumption increases mobile users’ message fatigue (Hickerson & Stamps, 2023). Individuals who engage with multiple media platforms (including digital newspapers, online news sites, and news apps on smartphones or tablets) are similarly more likely to experience exhaustion from repeated exposure to daily news, i.e., message fatigue (Andersen, 2022). Such repeated exposure may increase their level of exhaustion over time considering that Chinese users both actively and passively engage with a substantial amount of oral health-related content across various short-video apps on a daily basis. Therefore, the following hypothesis is proposed.

**Hypothesis** **3.** *EOHSV is positively associated with Chinese young adults’ message fatigue.*

Prior empirical evidence suggests that greater perceived health information fatigue among Chinese users is associated with increased information avoidance (Zhong & Gu, 2024). Similarly, during the COVID-19 pandemic, young adults’ pandemic-related exhaustion was positively linked to health information avoidance (Ford et al., 2023), indicating that fatigue induced by prolonged exposure to pandemic-related content further increased their tendency to avoid relevant disease and health information. News fatigue has also been identified as a key determinant of users’ information avoidance behaviors (M. Chan et al., 2024). Collectively, these findings provide empirical support for a mediating mechanism through which EOHSV may influence OHIA via message fatigue. The present study accordingly proposes the following mediation hypothesis.

**Hypothesis** **4.** *Message fatigue mediates the association between EOHSV and OHIA among Chinese young adults.*

### 2.4. Moderation Role of Loss Aversion

**Figure 1:**
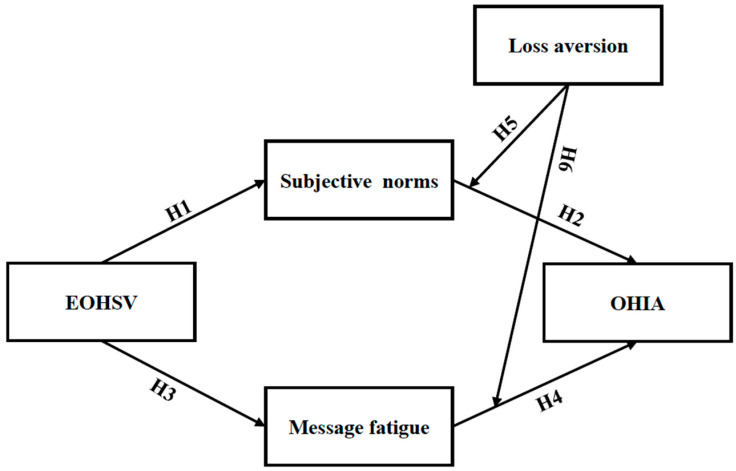
Conceptual model and hypothesized relationships of oral health information avoidance.

While prior research links social media engagement to information avoidance through normative and psychological mechanisms, cognitive biases, particularly loss aversion, may also serve as a moderating factor. For instance, prior research found that individuals with higher levels of loss aversion regarding cancer concerns were more likely to avoid receiving cancer-related information (Lam & Lin, 2025). Studies conducted during the COVID-19 pandemic similarly showed that individuals were more likely to avoid health information due to anticipated negative emotional responses, such as perceived infection risk, fear, and anxiety (Link, 2021). Such patterns are consistent with the mechanism of heightened loss aversion. As ongoing evidence suggests, individuals tend to defensively resist accepting recommended health behaviors when these are perceived as carrying greater psychological weight in terms of potential losses or emotional costs than anticipated gains (Orom et al., 2018). It is therefore plausible to argue that loss aversion functions as a moderating mechanism that conditions the association between message fatigue and oral health information avoidance among Chinese young adults. Despite this, this pathway has not yet been empirically examined. Therefore, the present study proposes the following hypotheses, and the hypothesized model is presented in Figure 1.

**Hypothesis** **5.** *Loss aversion positively moderates the association between subjective norms and OHIA among Chinese young adults*.

**Hypothesis** **6.** *Loss aversion positively moderates the association between message fatigue and OHIA among Chinese young adults*.

## 3. Materials and Methods

### 3.1. Questionnaire Design

Five established measures were adopted in the current study, namely, EOHSV, subjective norms, message fatigue, loss aversion, and OHIA from prior literature, and their items were adapted to reflect the Chinese context. Several steps were taken to ensure validity and cultural appropriateness. First, a back-translation method was applied: the questionnaire was originally developed in English and then translated into Chinese by professional translators (Guo et al., 2020). Second, content and face validity were carefully evaluated for each measure (Broder et al., 2007). Third, a pilot test was conducted with 15 volunteers to assess clarity and comprehensibility (Seo et al., 2016).

Data were collected between 10 December 2025, and 10 February 2026, via Tencent Survey (https://wj.qq.com; accessed on 10 December 2025), a widely used online survey platform in China for public health and health communication research (Lu et al., 2021). The finalized questionnaire was uploaded to the platform, and the survey link and QR code were distributed by the platform’s staff to respondents within its participant pool. Ethical approval was obtained from the Ethics Committee of the Beijing Institute of Graphic Communication (protocol code: IRB20251201), and the approval statement was included at the beginning of the questionnaire. Informed consent was presented on the first page, outlining the study’s purpose, procedures, and data anonymity. Participation was entirely voluntary, and participants were informed of their right to withdraw at any time without adverse consequences.

A total of 320 potential respondents received the survey link. After data cleaning, including the removal of speeders, 306 valid responses were retained for analysis. Accordingly, an a priori power analysis was conducted using G*Power 3.1.9.4 to determine the minimum required sample size. Following established recommendations for statistical power estimation, the analysis specified a power level of 0.85, an effect size of f^2^ = 0.15, and six predictors (four antecedents and two interactions). The results indicated a minimum required sample size of 109. As the final sample size of 306 exceeded this threshold, the study demonstrates adequate statistical power.

### 3.2. Measurement of Variables

All measures were assessed using a five-point Likert scale, with response options ranging from “strongly disagree” to “strongly agree” or from “very rarely” to “very frequently”, depending on the construct. EOHSV was measured using seven items adapted from the Social Media Engagement with Content Scale (Schivinski et al., 2016), selected based on factor loadings and conceptual relevance. EOHSV captures both passive engagement (e.g., viewing or watching content) and active engagement (e.g., liking, commenting, and saving). Sample items included “I have posted comments in the comment sections of these short videos,” “I have liked these short videos” and “I have saved these short videos” (M = 3.08, SD = 1.21, α = 0.94).

The subjective norms measure was adapted from Zou et al. (2023). This measure assessed Chinese young adults’ perceptions that important others expect them to avoid engaging with oral health-related information. Respondents indicated their level of agreement with four items using a five-point Likert scale. Sample items included “My family and friends expect me not to save oral health–related short videos” and “People important to me expect me to stop following and learning about oral health–related short-video content.” The scale demonstrated acceptable reliability (M = 2.50, SD = 1.08, α = 0.92).

The message fatigue scale was adapted from Song et al. (2017). The five-item measure assessed Chinese young adults’ feelings of exhaustion and boredom resulting from frequent engagement with excessive oral health-related short-form video content. Respondents indicated their level of agreement using a five-point Likert scale. Sample items included “I feel overwhelmed by the large number of short videos,” “I do not have enough time to keep up with these short videos,” and “The volume of these short videos exceeds what I can handle” (M = 3.11, SD = 1.08, α = 0.91).

The loss aversion scale was adapted from an existing measure (De Baets & Buelens, 2012). The scale assessed the extent to which respondents weighed the perceived costs or potential losses associated with adopting oral health practices more heavily than the corresponding benefits. Respondents indicated their level of agreement using a five-point Likert scale with four items. Sample items included “Changing my current oral health habits would lead to negative consequences,” and “Even if beneficial, changing my oral health practices involves risks” (M = 2.87, SD = 1.13, α = 0.89).

OHIA was adapted from Guo et al. (2020) to assess respondents’ intention to avoid general information on social media (Guo et al., 2020). The items from the original scale were modified to reflect the oral health context. The scale assessed individuals’ level of agreement with four statements using a five-point Likert scale. Sample items included “I intentionally ignore some oral health–related short videos,” “I do not want to follow such short videos,” and “When browsing short videos, I deliberately avoid oral health–related content.” The scale demonstrated acceptable internal consistency (M = 2.74, SD = 1.20, α = 0.94).

## 4. Results

### 4.1. Validity Tests

At the preliminary stage of data analysis, measurement validity was assessed. Confirmatory factor analysis (CFA) was conducted, discriminant validity was examined, and Harman’s one-factor test was performed to detect potential common method bias. These procedures were carried out to verify that the measurements used in this study adequately represented their intended constructs (Akbari et al., 2023; H. Liu et al., 2022; J. Yu et al., 2024).

CFA was conducted to examine whether the data adequately fit the hypothesized measurement model. Following prior methodological recommendations, a single-factor model, in which all items were constrained to load onto one latent factor, was also estimated to assess potential common method bias (H. Liu et al., 2022). The hypothesized multi-factor model demonstrated substantially better fit than the single-factor model. The analyses were performed using Jamovi 2.6.44. The results indicated an acceptable model fit, with χ^2^/df = 2.39, CFI = 0.95, TLI = 0.94, and RMSEA = 0.06 (90% CI: 0.06–0.07). The standardized factor loadings for each construct, together with composite reliability (CR) and average variance extracted (AVE), are presented in Appendix A. All constructs demonstrated CR values above 0.70 and AVE values above 0.50, indicating satisfactory convergent validity. Discriminant validity was evaluated using the Fornell–Larcker criterion (Akbari et al., 2023), with the square root of AVE for each construct exceeding its correlations with other constructs (see Table 1), thereby confirming adequate discriminant validity.

To further assess measurement validity, discriminant validity was evaluated using the heterotrait–monotrait ratio (HTMT), a correlation-based alternative approach (L. Zhang, 2022). The HTMT values for the five constructs are presented in Table 2. All values were below the recommended threshold of 0.85, indicating that discriminant validity is adequately established in this study.

As a final step to verify construct validity, Harman’s one-factor test was conducted to assess potential common method bias (J. Yu et al., 2024). The results showed that a single factor accounted for 41.10% of the total variance, which is below the recommended threshold of 50%. Accordingly, common method bias was not evident.

### 4.2. Descriptive Data

Based on valid responses (N = 306), the sample consisted of 174 females (56.9%) and 132 males (43.1%). In terms of age, 109 respondents were aged 18–23 years (35.6%), 97 were aged 24–29 years (31.7%), and 44 (14.4%) and 56 (18.3%) were aged 30–35 years. Regarding educational attainment, 47 respondents reported a high school education or lower (15.4%), 227 held a bachelor’s degree (74.2%), and 28 (9.2%) and 4 (1.3%) held a master’s degree or above. With respect to monthly income, 15 respondents (4.9%) earned RMB 1000–2999; 60 (19.6%) earned RMB 3000–4999; 106 (34.6%) reported RMB 5000–9999; 90 (29.4%) reported RMB 10,000–19,999; 25 (8.2%) reported RMB 20,000–39,999; and 10 (3.3%) reported RMB 40,000 or above.

### 4.3. Hypothesis Testing

#### 4.3.1. Path Analysis Tests

Prior to testing the direct hypotheses, the overall model fit was examined using the valid dataset. EOHSV and control variables (gender, age, education level, and income) were specified as exogenous variables, while subjective norms, message fatigue, and OHIA were treated as endogenous variables. The model demonstrated mixed fit: χ^2^/df = 4.10, *p* = 0.017, CFI = 0.99, GFI = 1.00, SRMR = 0.01, and RMSEA = 0.10 (90% CI: 0.04–0.18). While most fit indices met recommended thresholds, the RMSEA exceeded recommended thresholds, indicating possible model misspecification. Therefore, the results should be interpreted with caution. The model was employed for hypothesis testing.

The direct paths corresponding to Hypotheses 1 and 3 were examined. EOHSV was significantly associated with subjective norms among Chinese young adults; however, the association was negative (β = −0.77, z = −21.27, *p* < 0.001, 95% CI [−0.76, −0.63]), contrary to the hypothesized direction. Thus, Hypothesis 1 was rejected. EOHSV also showed a significant positive association with message fatigue (β = 0.44, z = 8.90, *p* < 0.001, 95% CI [0.31, 0.48]), indicating that more frequent engagement with oral health-related short-video content is associated with higher levels of exhaustion and boredom toward such health content. Accordingly, Hypothesis 3 was supported.

#### 4.3.2. Mediation Analysis Test

To examine the mediation hypotheses, SPSS 29.0 with the PROCESS macro (Model 4) was employed in the present study. A bootstrap procedure with 2000 resamples and 95% confidence intervals was used to estimate indirect association (Hou et al., 2020). Age, gender, education level, and income were included as control variables. The results indicated that subjective norms were positively associated with Chinese young adults’ OHIA (R^2^ = 0.43, β = 0.24, t = 3.12, *p* < 0.001, 95% CI [0.08, 0.39]). However, the indirect association was negative and significant (β = −0.16, t = 5.25, *p* < 0.001, 95% CI [−0.28, −0.05]). Hypothesis 2 was thus not supported. Additionally, message fatigue was found to be a key antecedent positively associated with OHIA (R^2^ = 0.43, β = 0.68, t = 12.02, *p* < 0.001, 95% CI [0.57, 0.79]). The indirect association of message fatigue in the relationship between EOHSV and OHIA was also significant (β = 0.27, t = 5.25, *p* < 0.001, 95% CI [0.19, 0.35]). Therefore, Hypothesis 4 was supported.

#### 4.3.3. Moderation Analysis Test

**Figure 2:**
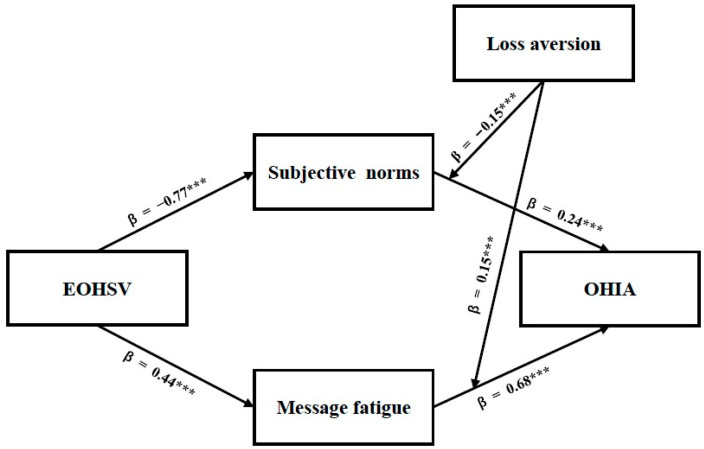
Results of the hypothesized relationships in oral health information avoidance. *** *p* < 0.001.

Moderation analysis was conducted using Hayes’ PROCESS macro (Model 1) in SPSS 29.0 (Kang & Park, 2025). Following the previous analytical procedure, the first moderation model indicated that loss aversion was significantly associated with OHIA as a main effect (R^2^ = 0.62, β = 1.13, t = 14.64, *p* < 0.001, 95% CI [0.98, 1.29]). In addition, the interaction between loss aversion and subjective norms on OHIA was significant and negative (β = −0.15, t = −4.83, *p* < 0.001, 95% CI [−0.21, −0.08]), indicating that loss aversion moderates the relationship between subjective norms and OHIA. This finding indicates that the interaction effect was opposite to Hypothesis 5. The second moderation model also indicated that loss aversion was not significantly associated with OHIA as a main effect (R^2^ = 0.65, β = 0.14, t = 1.18, *p* > 0.05, 95% CI [−0.09, 0.37]). However, the interaction between loss aversion and message fatigue on OHIA was significant (β = 0.15, t = 4.62, *p* < 0.001, 95% CI [0.08, 0.21]), indicating a significant moderating effect of loss aversion. Thus, Hypothesis 6 was supported. The results of the mediation and moderation analyses are presented in Table 3, and the hypothesized pathways are shown in Figure 2.

## 5. Discussion

### 5.1. Interpretation of Main Findings

Despite increasing efforts to promote health topics on popular social media platforms (i.e., short-form video apps) (Goldberg et al., 2022; Huang et al., 2025; Sharma et al., 2022; Y. Sun, 2025), a growing body of research has examined the association between health information engagement and users’ behavioral outcomes worldwide (Howell & Shepperd, 2017; Zhu et al., 2024). At the same time, an emerging phenomenon has been observed in which media users defensively avoid or disengage from health-related content (Howell et al., 2020; Sweeny et al., 2010). Oral health short videos represent one example of such content, and similar patterns have increasingly been observed in the Chinese social media environment, attracting growing scholarly attention. The antecedents and underlying mechanisms of oral health information avoidance nevertheless remain relatively underexplored. Guided by Prospect Theory, the present research examines how engagement with oral health short videos, together with key psychological and normative drivers, contributes to oral health information avoidance among Chinese young adults. Specifically, parallel mediating associations of subjective norms and message fatigue are proposed, along with the moderating role of loss aversion. The key findings are discussed in this section.

Prior health communication research suggests that engagement with media content can strengthen individuals’ subjective norms (Cristello et al., 2024). For example, exposure to alcohol and drug prevention campaigns has been positively associated with perceived social expectations regarding these behaviors (Cristello et al., 2024). However, the present study reveals a reversed pattern. Specifically, engagement with oral health short videos was negatively associated with subjective norms among Chinese young adults, indicating that more frequent engagement is linked to weaker perceptions that significant others expect them to avoid such content. One possible explanation lies in social endorsement cues within social media environments (Kim, 2021). Content with greater visibility through likes, shares, and comments is more likely to be perceived as socially approved and credible (Borah & Xiao, 2018). In this context, such endorsement may reduce perceived social pressure to avoid oral health content, thereby weakening subjective norms that discourage engagement. Despite the inconsistent findings for Hypothesis 1, a positive association between engagement with oral health short videos and message fatigue among Chinese young adults was evident. The results also indicated a relatively strong association (β = 0.44, z = 8.90, *p* < 0.001, 95% CI [0.31, 0.48]). This finding is consistent with prior research suggesting that frequent engagement with media platforms can increase individuals’ feelings of exhaustion due to repeated exposure to similar health-related messages (Hickerson & Stamps, 2023). This pattern is also plausible in the contemporary Chinese media environment, where young adults both actively and passively encounter large volumes of oral health-related content through short-video platforms on a daily basis, and such repeated exposure can increase feelings of exhaustion over time.

Unexpectedly, the first indirect association revealed a reversed pattern, indicating that subjective norms were negatively associated with avoidance behavior in the relationship between engagement with oral health short videos and avoidance among Chinese young adults. This finding contradicts the theoretical expectation derived from the Theory of Planned Behavior, which posits that subjective norms typically reinforce behavioral intentions in alignment with perceived social expectations (Cristello et al., 2024). This inconsistency suggests a potential context-specific deviation from TPB assumptions. Although prior research has linked stronger subjective norms to disengagement from digital platforms (Shittu & Salisu, 2023), the direction and implications of such norms may vary across contexts. Therefore, these findings should be interpreted with caution.

To provide additional context for this unexpected pattern, exploratory follow-up interviews were conducted between 25 February and 10 March 2026 with six participants who had indicated willingness to participate, using a convenience sampling approach. Interviews were conducted online via Tencent Meeting. Participants were informed of study procedures and ethical considerations, and anonymity was assured. A semi-structured format was employed, and the data were analyzed thematically to identify recurring patterns. Participants were asked, “To what extent do the opinions and social expectations of people around you influence your willingness to watch or engage with oral health short videos?” Four participants reported prioritizing social expectations over their own judgments. In some cases, such expectations appeared to heighten perceived threat and concern regarding oral diseases. For example, one participant noted, “I care about the opinions of my closest friends and family members when it comes to oral care… this makes me worry about whether I might have, or will develop, an oral disease” (M1). Another stated, “My friends once emphasized oral health risks, which made me feel worried and somewhat fearful” (F3). These findings provide preliminary interpretive context for the quantitative results. Rather than serving as confirmatory evidence, they suggest a potential mechanism whereby social expectations may heighten perceived threat and may be linked to reduced avoidance. However, given the small, non-representative sample and the post hoc design, these findings should be interpreted with caution. The second mediation result indicates that message fatigue emerged as a key mediator in the relationship between frequent engagement with oral health short videos and avoidance behavior. This finding is consistent with prior research showing that message fatigue is indirectly associated with social media engagement and message avoidance behavior (M. Chan et al., 2024; Ford et al., 2023; Hickerson & Stamps, 2023).

Moderation analyses were also conducted. Prior research suggests that online media users who weigh potential losses more heavily than anticipated gains (i.e., loss aversion) are more likely to resist adopting recommended health behaviors (Lam & Lin, 2025). Loss aversion positively moderated the association between message fatigue and oral health information avoidance in this study (β = 0.15, t = 4.62, *p* < 0.001, 95% CI [0.08, 0.21]). In other words, the positive interaction indicates that the association between message fatigue and oral health information avoidance becomes stronger among individuals with higher levels of loss aversion. Guided by Prospect Theory, this study provides initial empirical evidence that the interaction between loss aversion and message fatigue is associated with oral health information avoidance among young adults in the Chinese context.

### 5.2. Theoretical Implications

This study makes a threefold theoretical contribution. First, by integrating insights from Prospect Theory and TPB, the findings support the explanatory role of loss aversion in shaping oral health information avoidance among Chinese young adults, extending its application beyond Western contexts. Second, the results contribute to TPB by clarifying the role of subjective norms as perceived social expectations that discourage engagement with oral health-related information, representing an application of TPB to behavioral inhibition. Importantly, these findings suggest that individual differences in loss aversion (Prospect Theory) may condition how social expectations (TPB) are translated into behavioral responses. Third, loss aversion positively moderates the relationship between message fatigue and avoidance behavior, indicating that individuals with higher sensitivity to potential losses are more likely to translate fatigue into avoidance. This highlights its role as a boundary condition shaping the strength of behavioral responses.

### 5.3. Practical Implications

Based on the findings, engagement with oral health short videos is associated with increased message fatigue among Chinese young adults, likely driven by excessive algorithm-based exposure that is misaligned with users’ needs. Regulating content frequency and implementing personalized delivery systems are accordingly essential to mitigating fatigue and sustaining engagement (J. Sun et al., 2025). In this regard, recommendation and push-based information delivery should be applied cautiously to avoid excessive or redundant exposure. This study also suggests that practitioners and content creators should optimize message framing by reducing reliance on loss or threat-based appeals and incorporating gain-framed, solution-oriented, and supportive messages. For example, content can emphasize the benefits of good oral hygiene (e.g., fresher breath and improved dental health) and provide actionable guidance, such as proper brushing techniques and regular dental check-ups, to enhance message receptivity. Furthermore, AI-generated health content is becoming increasingly popular among young adults in China, reflecting their strong interest in such content (M. Chan et al., 2026); therefore, health communication influencers and content creators on social media platforms should leverage AI-based video tools to produce high-quality, educational, and less anxiety-provoking materials, which may improve engagement and acceptance.

### 5.4. Limitations

The present study has several limitations. First, the sample is relatively narrow, focusing exclusively on Chinese young adults aged 18–35. Although this group represents a primary user base of short-form video platforms, other age groups are also increasingly engaged with online oral health information. Future research should therefore include a broader age range to improve generalizability. Second, although guided by Prospect Theory, this study examines loss aversion as an individual difference factor without manipulating gain–loss framing, limiting direct assessment of the theory. Future research should employ experimental designs to address this issue. Finally, although Harman’s single-factor test was conducted, common method bias cannot be entirely ruled out due to the use of self-reported, single-source data. Accordingly, the findings should be interpreted with caution, and future studies are encouraged to adopt multi-source or longitudinal designs.

## 6. Conclusions

An emerging body of research has identified health information avoidance as a notable behavioral response among young adults. Within this context, oral health-related information represents a domain in which individuals may exhibit reluctance to engage with persuasive messages. Guided by Prospect Theory, this study examines the association between engagement with oral health short videos and information avoidance among Chinese young adults, with subjective norms and message fatigue as mediators and loss aversion as a moderator. A cross-sectional survey was conducted to test the proposed model. This study advances understanding of the psychological mechanisms underlying oral health information avoidance and contributes to health communication research. The findings also offer practical implications. Excessive algorithm-driven exposure may contribute to message fatigue. Therefore, regulating content frequency and implementing personalized delivery systems, along with the cautious use of recommendation and push-based strategies, is essential. Optimizing message framing by reducing reliance on loss-based appeals and incorporating gain-framed, solution-oriented content may also improve engagement and acceptance. Finally, leveraging AI-based video tools to deliver high-quality, educational, and less anxiety-provoking content may further enhance communication effectiveness.

## Figures and Tables

**Table 1:** Results of the Fornell–Larcker criterion for discriminant validity (n = 306).

Factors	1	2	3	4	5
EOHSV	0.85				
Subjective norms	−0.77 **	0.89			
Message fatigue	0.44 **	−0.38 **	0.85		
Loss aversion	0.27 **	−0.16 **	0.61 **	0.86	
OHIA	0.28 **	−0.16 **	0.63 **	0.76 **	0.92

** *p* < 0.01, EOHSV = engagement with oral health short videos; OHIA = oral health information avoidance.

**Table 2:** Results of the HTMT for discriminant validity (n = 306).

Factors	1	2	3	4	5
EOHSV					
Subjective norms	0.83				
Message fatigue	0.47	0.41			
Loss aversion	0.29	0.18	0.68		
OHIA	0.30	0.17	0.68	0.83	

EOHSV = engagement with oral health short videos; OHIA = oral health information avoidance.

**Table 3:** Results of the mediation and moderation analyses (n = 306).

Pathways	β	t	*p*	LLCI	ULCI
Mediation analysis					
EOHSV → subjective norms → OHIA	−0.16	5.25	*p* < 0.001	−0.28	−0.05
EOHSV → message fatigue → OHIA	0.27	5.25	*p* < 0.001	0.19	0.35
Moderation analysis					
Loss aversion × subjective norms → OHIA	−0.15	−4.83	*p* < 0.001	−0.21	−0.08
Loss aversion × message fatigue → OHIA	0.15	4.62	*p* < 0.001	0.08	0.21

EOHSV = engagement with oral health short videos; OHIA = oral health information avoidance.

## Data Availability

The original data are provided by all the authors. If there are relevant research needs, the data can be obtained by sending an email to the corresponding author. Please indicate the purpose of the research and the statement of data confidentiality in the email.

## Abbreviations

The following abbreviations are used in this manuscript:

EOHSV	Engagement with oral health short videos
OHIA	Oral health information avoidance
CFA	Confirmatory factor analysis
CR	Composite reliability
AVE	Average variance extracted
TPB	Theory of Planned Behavior

## Appendix A

**Table A1:** Results of the measurement model (factor loadings, CR, and AVE).

Construct	Items	Factor Loadings	CA	CR	AVE
EOHSV	EOHSV1	0.77	0.94	0.94	0.73
	EOHSV2	0.85			
	EOHSV3	0.90			
	EOHSV4	0.89			
	EOHSV5	0.81			
	EOHSV6	0.84			
	EOHSV7	0.88			
Subjective norms	SN1	0.84	0.92	0.94	0.80
	SN2	0.91			
	SN3	0.93			
	SN4	0.88			
Message fatigue	MF1	0.80	0.91	0.93	0.73
	MF2	0.77			
	MF3	0.89			
	MF4	0.89			
	MF5	0.90			
Loss aversion	LA1	0.88	0.89	0.92	0.75
	LA2	0.89			
	LA3	0.84			
	LA4	0.85			
OHIA	OHIA1	0.91	0.94	0.95	0.85
	OHIA2	0.92			
	OHIA3	0.93			
	OHIA4	0.91			

EOHSV = engagement with oral health short videos; OHIA = oral health information avoidance.
